# Effect of Inflammatory Mediators on ATP Release of Human Urothelial RT4 Cells

**DOI:** 10.1155/2014/182862

**Published:** 2014-04-15

**Authors:** Kylie J. Mansfield, Jessica R. Hughes

**Affiliations:** Graduate School of Medicine, Illawarra Health and Medical Research Institute, University of Wollongong, Wollongong, NSW 2522, Australia

## Abstract

Inflammation is an important contributor to the aetiology of a number of bladder dysfunctions including interstitial cystitis, painful bladder syndrome, and overactive bladder. The aim of this study was to examine the effects of inflammatory mediators on urothelial ATP release. Human urothelial RT4 cells were exposed to normal buffer or varying concentrations of inflammatory mediators (bradykinin, histamine, and serotonin) in the presence or absence of hypotonic stretch stimuli (1 : 2 dilution of Krebs-Henseleit buffer). Others have demonstrated that bradykinin increased stretch-induced ATP release; however, we observed no change in control or stretch-induced ATP release with bradykinin. Pretreatment of RT4 cells with histamine or serotonin decreased stretch-induced ATP release (*P* = 0.037, *P* = 0.040, resp.). Previous studies have demonstrated increased ATP release in response to inflammation utilising whole bladder preparations in contrast to our simple model of cultured urothelial cells. The current study suggests that it is unlikely that there is a direct interaction between the release of inflammatory mediators and increased ATP release, but rather more complex interactions occurring in response to inflammation that lead to increased bladder sensation.

## 1. Introduction

Inflammation is an important contributor to the aetiology of a number of bladder dysfunctions. Pyuria (the presence of white blood cells in the urine) has been associated with lower urinary tract symptoms [1] and biopsy specimens from patients with interstitial cystitis (IC)/painful bladder syndrome (PBS) are commonly characterised by the presence of inflammatory cells in the lamina propria, especially infiltration with mast cells [2–7]. Recently, histological evidence for chronic inflammatory infiltrate has been demonstrated in patients with refractory overactive bladder (OAB) [8] together with pyuria in these patients [8, 9]. Proinflammatory cytokines are increased in the urine from patients with OAB [10–12].

These conditions (IC/PBS/OAB) are all characterised by urinary urgency, together with frequency and nocturia [13]. It is believed that bladder sensation is associated with the interaction of ATP with purinergic receptors located on suburothelial afferent nerves [14, 15] and myofibroblasts [15]. ATP is released from the urothelium in response to stretch of the urothelium triggered by bladder filling [16]. An increase in stretch-induced ATP release has been demonstrated in tissue strips and biopsies from patients with IC/PBS [17–19] and OAB [20]. In addition, the concentration of ATP in intravesical fluid has been shown to correlate with urinary urgency as indicated by the volume at first desire to void in patients with OAB [21, 22].

Similar to the changes in bladder histological structure described with PBS/IC, chemical-induced cystitis causes histological changes in the bladder wall including infiltration of inflammatory cells (e.g., mast cells and macrophages) into the submucosa [4–7], together with increased bladder weight and oedema. In addition, chemical cystitis is associated with activation of previously silent C-fibre afferents and sensitisation of mechanosensitive A*δ*-fibres within the submucosa [23–26].

The effect of inflammation causing an increase in urothelial ATP release has been examined in animal models of inflammation, including feline interstitial cystitis [27], cyclophosphamide cystitis [28], and ketamine cystitis [2]. However, the agents responsible for the increased ATP release are unknown. The aim of this study was to examine the effects of bradykinin, histamine, and serotonin, mediators commonly associated with bladder inflammation, on urothelial cell ATP release.

## 2. Materials and Methods

### 2.1. Cell Culture

Human urothelial RT4 cells (obtained from the ECACC) were grown at 37°C with 5% CO_2_ in McCoy's 5A culture medium supplemented with 10% foetal bovine serum, 100 units/mL of penicillin, 100 *μ*g/mL of streptomycin, and 0.25 *μ*g/mL of fungizone. At confluence, cells were passaged and then replated onto T75 flasks for continuous passage or onto 24 well plates for use in ATP release when confluent (approximately 3 to 5 days after passage).

### 2.2. ATP Release

ATP release was determined as previously described [29]. Urothelial cells were washed (three times) with carbogenated Krebs-Henseleit solution (containing mM: NaCl 118, KCl 4.7, NaHCO_3_ 25, KH_2_PO_4_ 1.2, MgSO_4_ 1.2, CaCl_2_ 2.5, and D-glucose 11.7). The basal level of ATP release was then determined by 10-minute incubation in 500 *μ*L Krebs-Henseleit solution. Cells were then exposed to normal Krebs-Henseleit (control) or the indicated concentration of mediator (bradykinin, histamine, and serotonin) in the presence or absence of hypotonic Krebs-Henseleit (1 : 2 dilution of Krebs-Henseleit in distilled water). Hypotonic Krebs-Henseleit was used as a stretch stimulus to examine the effect of the mediators on stretch-induced ATP release. Cells were treated for 10 min before the supernatant (200 *μ*L) was collected and used for ATP determinations.

ATP concentration in the supernatant was measured using the bioluminescence assay. Equal volumes of the cellular supernatant or ATP standard solutions (10^−6^ to 10^−10^ M) were mixed with the bioluminescence assay mix and the luminescence generated was measured immediately using a plate reader (BMG Labtech Polarstar). The ATP concentration in the cell supernatant was calculated relative to the standard curve. Treatments were carried out in triplicate and the mean ATP concentration (in nM) per treatment was determined.

### 2.3. Statistics

As results are nonnormally distributed and as such are expressed as median with interquartile range (IQR); two different treatments were compared using a Wilcoxon matched-pairs *t*-test. Dose response relationships were examined using a sigmoidal dose response curve. All statistics were performed using Graphpad Prims (version 6) (San Diego, CA).

### 2.4. Materials

All cell culture reagents were purchased from Invitrogen (Mount Waverley, Australia). Bioluminescence ATP assay kit and mediators (bradykinin, histamine, and serotonin) were from Sigma-Aldrich (Sydney, Australia). All other reagents were of high analytical grade.

## 3. Results and Discussion

### 3.1. Effect of Bradykinin on Urothelial Cell ATP Release

Bradykinin is a peptide neurotransmitter released from sensory afferent nerves. Bradykinin release is closely associated with inflammatory responses. Distension of the bladder has been shown to stimulate release of bradykinin in patients with IC indicating a potential role for this peptide in the pathophysiology of this disorder [30]. Bradykinin exerts its physiological actions via activation of B1 and B2 receptors. Bradykinin B2 receptors are believed to be important in inflammatory pain with animal models showing that block of this receptor reduces inflammatory hyperalgesia [31, 32]. In contrast, bradykinin B1 receptors are expressed at low levels under normal circumstances with their expression upregulated following tissue damage or inflammation [33]. This includes upregulation of bradykinin B1 receptors following cystitis induced by intravesical injection of the detergent Triton X100 [34]. Expression of bradykinin B1 receptors is increased in biopsies obtained from patients with interstitial cystitis [35] and, in cyclophosphamide-induced cystitis, B1 receptor mediated bladder responses are significantly increased [36, 37]. In addition, cyclophosphamide cystitis induced a bladder hyperactivity that was dependent on bradykinin B2 receptor activation and was inhibited by the P2 receptor antagonist PPADS [38]. This indicates a role for both bradykinin and ATP in cyclophosphamide-induced cystitis [38].

**Figure 1:**
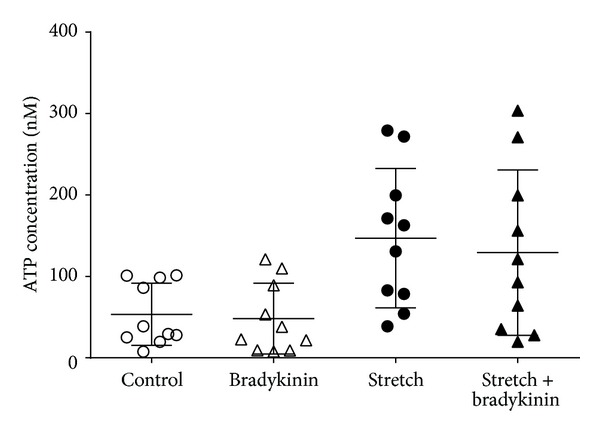
Effect of bradykinin (1 *μ*M, *n* = 10) on baseline level of ATP release and release induced by hypotonic media. Symbols are representative of individual data points for the four groups. Data are shown as median with interquartile range.

Other studies have observed that activation of bradykinin B2 receptors by bradykinin increases stretch-induced ATP release in UROtsa urothelial cells [39] and in primary cultures of rat urothelial cells [38]. However, in the current study, using urothelial RT4 cells, we saw no change in either control or stretch-induced ATP release in the presence of bradykinin (Figure 1). Hypotonic Krebs which was used as a positive control was seen to induce an approximate threefold increase in ATP release (baseline ATP release 34.06 (23.8–99.1) Nm; stretch-induced ATP release 146.7 (72.4–217.6) nM, *P* = 0.002). It is possible that the differences in these findings relate to the individual cell lines used and that the bradykinin receptors usually present on urothelial cells are not functional on RT4 urothelial cells.

### 3.2. Effect of Mast Cell Mediators on Urothelial Cell ATP Release

Infiltration of inflammatory cells such as mast cells and macrophages into the bladder submucosa has been demonstrated in cyclophosphamide-induced cystitis [3] and ketamine-induced cystitis [2]. In addition, it is well known that there is an increased density of mast cells in the bladder wall in patients with IC [4–7]. Mast cells lie in close proximity to both urothelial cells and afferent fibres in the submucosa of the urinary bladder, and degranulation of these cells has been shown to release a wide range of neurotransmitters and cytokines [38].

Mast cells are granulated immune system cells which make up the major sensory arm of the innate immune system [40]. Mast cells respond to allergens as well as nonimmunologic stimuli such as bacteria, chemicals, kinins, and neuropeptides [41] to release mediators such as histamine and serotonin [42]. There are reports of increased concentrations of histamine in urine from IC patients [41] and it is believed that the pain associated with bladder filling in IC is related to release of histamine from mast cells in the bladder wall [43].

**Figure 2:**
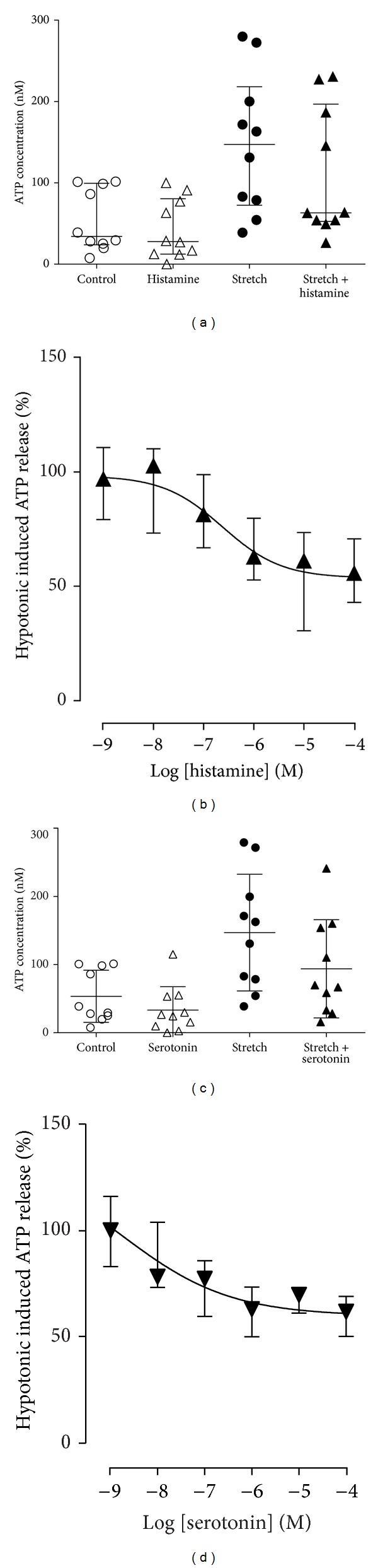
Effect of histamine (1 *μ*M) ((a), *n* = 10) and serotonin (1 *μ*M) ((c), *n* = 10) on baseline level of ATP release and release induced by hypotonic media. Data are shown as median with interquartile range. Concentration response relationships for the inhibition of ATP release induced by hypotonic media in the presence of histamine ((b), *n* = 10) and serotonin ((d), *n* = 10).

In this study, the effect of mast cell mediators, histamine and serotonin, on urothelial cell ATP release was examined. Incubation of the RT4 cells with mast cell mediators, histamine and serotonin, for 10 minutes had no effect on control ATP release (Figures 2(a) and 2(c)). However, pretreatment of urothelial cells with histamine or serotonin for a 10-minute period prior to the addition of the hypotonic stretch stimulus resulted in a decrease in ATP release in response to stretch (Figure 2(a), *P* = 0.037 for histamine; Figure 2(c), *P* = 0.040 for serotonin). When the concentration response effect of histamine on stretch-induced ATP release was determined, it was seen to occur with an IC50 of 0.24 *μ*M (0.19–2.8 *μ*M) (Figure 2(b), *n* = 6). This concentration is within the histamine concentration range that has been reported in urine from IC patients (3 to 31 ng/mL, equating to 24 to 280 nM) [43]. The slope of the concentration response curve was fairly shallow with a Hill slope of −0.74 ± 0.19. A similar concentration dependent inhibition of ATP release was also elucidated for serotonin. In this instance, an IC50 of 1.7 nM (<0.001 nM–50.0 *μ*M) was determined with a Hill slope of −0.37 ± 0.24 (Figure 2(d), *n* = 6).

While the effect of the mast cell mediators on urothelial cell ATP release has never previously been examined, the results obtained were somewhat unexpected. Previous studies have shown an increase in ATP release from the bladder in response to inflammation [17–19, 27, 28]. However, all of these studies were conducted in whole tissue models of inflammation. In these models, determining the causative molecule or cell is complex as numerous interactions between different cell types and mediators are occurring in the one system which could change receptor expression in addition to mediator release in response to the inflammatory stimulus. The model used in the current study of cultured urothelial cells is a much simpler model containing only a single cell type and a single exogenously supplied mediator. It is possible that in the previous studies it was not the inflammatory mediators themselves that were inducing the increase in ATP release, but rather secondary changes that occurred in response to the inflammation. Decreasing importance of ATP in inflamed bladder is supported by findings that the P2 antagonist (PPADS) decreased voiding frequency in control animals but not in cyclophosphamide treated animals [44].

Inflammation causes a number of changes in the bladder. For example, cyclophosphamide induced an increase in COX2 protein expression in the urothelium, which was dependent on release of mediators from mast cells [45]; in addition, cyclophosphamide also increases prostaglandin E_2_ production [46]. Prostaglandin E_2_ has been shown to increase urothelial cell ATP release [47]. It is also possible that in response to inflammation there are changes in receptor expression that would alter the response of urothelial cells to these mast cell mediators. As mentioned previously, bradykinin B1 receptors are expressed at very low levels under normal conditions but are upregulated following inflammation [38]. It is possible that a similar phenomenon occurs for histamine and serotonin receptors on the urothelium although this has not been examined to date.

In addition, there are further changes that occur in the inflamed bladder including defects in urothelial junction proteins, increased suburothelial inflammation, and increased urothelial cell apoptosis [2]. Bladder inflammation is associated with disruption of the glycosaminoglycan layer that covers the urothelium [48–50]. Disruption of this layer may allow urine contents to evoke irritation and inflammation within the bladder wall. There is also increased density of submucosal afferent nerves in the inflamed bladder [43, 51, 52]. Urinary nerve growth factor (NGF) levels are increased in patients with IC [53] and OAB [54]. In addition, cyclophosphamide-induced cystitis is associated with increased NGF mRNA levels in the bladder [55], increases the density of afferent nerves, and increases expression of P2X_2_ and P2X_2/3_ receptors in afferent nerves [56]. It is likely that the increase in NGF expression in patients with bladder dysfunction is associated with the increased density of suburothelial nerves in these patients. NGF is also thought to alter expression of neurotransmitters, modulate ion channels, and increase excitability of afferent nerves [57]. In rats, exogenously applied intravesical NGF can induce bladder nociceptive responses and bladder overactivity [58]. In addition to NGF, inflammatory mediators themselves may sensitise the afferent nerves to the effects of ATP [56, 59], which could increase the bladder sensations in response to bladder filling in the inflamed bladder.

## 4. Conclusions

It is well accepted that bladder inflammation is associated with increased signalling from the bladder and increased sensation. Studies using whole bladder models of inflammation have shown that this is due to increased release of ATP from the urothelium. However, based on the results of the current study, it appears unlikely that there is a simple interaction between the release of the inflammatory mediators (bradykinin, histamine, and serotonin) and increased ATP release leading to increased bladder sensation. It is more likely that there are numerous factors involved in the interaction between bladder inflammation and increased sensation. These factors include alterations to receptor expression on the urothelium and afferent nerves, increased density of afferent nerves, and hyperresponsiveness of the afferent nerves, altering the neuronal component of afferent signalling.
